# Sudden development of adult-onset type II citrullinemia after total gastrectomy: a case report

**DOI:** 10.1186/s40792-018-0420-9

**Published:** 2018-01-25

**Authors:** Ryuji Komine, Keisuke Minamimura, Akihiro Watanabe, Atushi Shimizu, Kazuhiko Mori, Toru Hirata, Takashi Kobayashi, Sotaro Akatsuka

**Affiliations:** 1 Department of Gastrointestinal and General Surgery, Mitsui Memorial Hospital, 1, Izumi-cho, Kanda, Chiyoda-ku, Tokyo, 101-8643 Japan; 2 Department of Medical Oncology, Mitsui Memorial Hospital, 1, Izumi-cho, Kanda, Chiyoda-ku, Tokyo, 101-8643 Japan

**Keywords:** Adult-onset type II citurullinemia, Total gastrectomy, Conservative treatment, Liver transplantation

## Abstract

**Background:**

Adult-onset type II citurullinemia is an autosomal recessive disorder characterized by recurrent encephalopathy with hyperammonemia resulting from high plasma citrulline and ammonium levels. This report describes a rare case of adult-onset type II citurullinemia that occurred in a patient who only had the heterozygote mutation, and had never presented with any symptoms before surgery.

**Case presentation:**

A 56-year-old man underwent a total gastrectomy for stomach cancer. On postoperative Day 13, he suddenly developed presyncope, and blood tests showed hyperammonemia and high levels of serum citrulline. He was diagnosed with hepatic encephalopathy. DNA analysis revealed a heterozygote mutation in Solute Carrier Family 25. Although the patient received a conservative treatment, episodes of loss of consciousness and abnormality of behavior repeatedly occurred.

**Conclusion:**

Abdominal surgery involving the reconstruction of digestive tract alters the mechanisms of absorption and/or metabolism such that the symptoms of adult-onset type II citurullinemia may arise. Liver transplantation should be performed if all conservative treatments are unsuccessful.

## Background

Citrin deficiency is an autosomal disorder, and symptoms present during the childhood of most patients. Adult-onset type II citrullinemia 2 (CTLN2) is caused by mutations in the Solute Carrier Family 25 (*SLC25A13*) gene, which encodes a mitochondrial aspartate glutamate carrier protein. The symptoms of CTLN2 are often provoked by triggers such as alcohol, sugar intake, and surgery [1]. We report a rare clinical case of CTLN2, in which the patient had never experienced epileptic seizures or disturbances of consciousness until he underwent on a total gastrectomy.

## Case presentation

A laparoscopic-assisted total gastrectomy (final stage UICC 7th edition: T3N1M0, IIIB) was performed on a 56-year-old man to treat stomach cancer. On postoperative Day 13, the patient suddenly lost consciousness. Computed tomography showed no cerebral infarction and/or bleeding, and magnetic resonance imaging revealed no cerebral vascular accident, brain infarction, or encephalitis. Blood tests showed hyperammonemia (serum ammonia [NH3], 240 μg/dL; Table 1). He was diagnosed with hepatic encephalopathy and branched-chain amino acids (BCAAs) were administered. Two days after BCAAs administration, the serum NH3 level was reduced to 10% of hyperammonemic levels, and recovery of consciousness was observed. However, he was in an aggressive mental state for several days after recovery.

**Table 1:** Summary of the serum ammonia level and state of consciousness

	NH3(μg/dL)	State of consciousness	Diet before symptom onset	BCAAs
Days after operation				
13	478	Coma	Ordinary	Nothing
15	52	Alert	NPO	i.v.
Days after adjuvant chemotherapy				
14	46	Aberrant behaviors	Ordinary	Nothing
28	858	Coma	Ordinary	Nothing
29	93	Coma	NPO	i.v.
30	51	Alert	NPO	i.v.
Days after starting nutritional therapy				
1	NM	Alert	NPO	Nothing
7	237	Coma	Low-carbohydrate, high-protein/fat	i.v.
8	156	Aberrant behaviors	NPO	i.v.
11	442	Coma	Low-carbohydrate, high-protein/fat	i.v.
18	13	Alert	NPO	Nothing
27	47	Alert	Low-carbohydrate, high-protein/fat	Nothing
28	478	Coma	Low-carbohydrate, high-protein/fat	i.v.
29	52	Drowsiness	NPO	i.v.
30	12	Alert	Unbalanced	p.o.
36	148	Aberrant behaviors	Unbalanced	p.o.
37	NM	Alert	Unbalanced	p.o.
39	125	Drowsiness	Unbalanced	p.o.
42	NM	Alert	Unbalanced	p.o.
44	147	Drowsiness	Unbalanced	p.o.
45	NM	Alert	Unbalanced	p.o.

*NM* Not measurement, *NPO* Nothing per os, *i.v.* Intravenous injection, *p.o.* Per os

Unbalanced diet means taking only nut, cheese, fish, and small amout of carbohydrate

One month later, he received adjuvant chemotherapy (capecitabine + oxaliplatin) for gastric cancer. Two weeks after the start of the treatment, he experienced relapse of behavior disorder (serum NH3, 46 μg/dL). Electroencephalography demonstrated triphasic waves, accompanied with somewhat abnormal waves of metabolic abnormality. Two weeks later, he represented with loss of consciousness. Laboratory tests showed hyperammonemia (serum NH3, 858 μg/dL), and amino acid analysis showed hypercitrullinemia. Genomic DNA was extracted from his peripheral blood, and gene analysis revealed that he had a heterozygous 851del4 DNA nucleotide mutation in *SLC25A13* (solute carrier family 25 member 13). Although the mutation was heterozygous, he was diagnosed with CTLN2 based on the mutation, recurrent symptoms, hyperammonemia, and hepatic encephalopathy.

Before surgery, the patient preferred eating nuts and fresh cream, but disliked sweat bean paste. However, his habitual dietary intake ceased after surgery, and he began to eat carbohydrate-rich foods such as noodles.

Although the patient received nutritional therapy with a carbohydrate-restricted, high protein/fat diet (total calories, 1800 kcal/day; protein: fat: carbohydrate ratio, 15:40:45), hepatic encephalopathy and/or aberrant behavior repeatedly developed twice in a week. Thereafter, the patient started dietary treatment with BCAAs, without receiving any other therapy such as arginine or sodium pyruvate administration. Finally, he was required to return to his normal eating, which consisted of nuts, cheese, fish, and small amount of carbohydrates. Although the frequency of hepatic encephalopathy and/or aberrant behavior episodes decreased, the symptoms still presented occasionally. Additionally, despite being nondiabetic and having a normal blood glucose level before the total gastrectomy, hyperglycemia (> 200 mg/dL) was sometimed observed in the patient after he developed CTLN2 symptoms. Liver transplantation, which is the only radical treatment for CTLN2, will be considered should his condition worsen.

### Discussion

Citrin, a mitochondrial solute carrier protein encoded by *SLC25A13*, plays an important role in ureagenesis and gluconeogenesis. Citrin deficiency causes neonatal intrahepatic cholestasis (NICCD), failure to thrive and dyslipidemia caused by citrin deficiency (FTTDCD), and adult-onset type II citrullinemia (CTLN2) [1]. Citrin deficiency had been regarded as a hereditary disease in East Asia, but non-Asian cases have recently been reported. CTLN2 is an autosomal recessive disorder characterized by hyperammonemia and high plasma citrulline levels, accompanied by recurrent encephalopathy, delirium, abnormal behavior, seizures, and coma. The reduction of argininosuccinate synthetase activity in the urea cycle is regarded to cause of the symptoms [2–6].

Saheki and Kobayashi reported that approximately 1 in 70 people in Japanese population carry a mutation in one allele of the *SLC25A13* [7]. Though the minimal estimated frequency of homozygotes (or compound heterozygotes) for *SLC25A13* pathogenic variants is calculated to be 1 in 20,000, the incidence of CTLN2 is much lower, at 1 in 100,000 to 1 in 230,000 [1]. On the other hand, there are twice as many male CTLN2 patients as there are female, and such sex differences are not observed in NICCD patients [8]. There is a possibility that sex hormones affect enzymatic activity in the urea cycle, and can trigger CTLN2 symptoms even in patients with heterozygous mutations.

Diet also contributes to the occurrence of symptom, and patients tend to select an unbalanced protein- and lipid-rich diet. Komatsu et al. reported a correlation between the severity of hepatic steatosis and the down-regulation of free acid oxidation and proliferator-activated receptor alpha (PPARα), indicating that CTLN2 patients have low hepatic ATP levels; therefore, they prefer lipid-rich diets to generate more ATP through free acid oxidation [9].

Currently, conservative therapy for CTLN2 typically consists of a lipid- and protein-rich, low-carbohydrate diet, which may enhance mitochondrial β-oxidation activity and increase hepatic ATP levels [9]. However, sudden-onset symptoms, such as loss of consciousness and abnormal behavior, may be related to argininosuccinate synthase activity. Although administration of arginine and sodium pyruvate is also reported to improve hyperammonemia and delay the need for liver transplantation [10–13], the mechanism of action of this treatment remains unclear.

Alcohol, sugar intake, medication, and surgery are reported as precipitating factors of CTLN2 [1]. To the best of our knowledge, there are only two other cases reported in which CTLN2 occurred after surgical treatment (Table 2). One case was diagnosed at few days after total colectomy [14], while the other case was diagnosed 2 years after pancreaticoduodenectomy for duodenal malignant somatostatinoma [15]. Here, we report the third known case, diagnosed at 13 days after a total gastrectomy for stomach cancer as hepatic encephalopathy. The former two cases underwent liver transplantation after diagnosis of CTLN2. Typical conservative treatment consisting of nutritional therapy with a calorie/carbohydrate-restricted and protein-rich diet, was not effective in our case; the symptoms have occasionally recurred even after the patient returned to his normal eating habits. Reconstruction of the digestive tract is a common factor among these three patients, and indicates that the change in the mechanisms of nutrient absorption and/or metabolism may be a trigger of CTLN2. To supplement this notion, the plasma insulin level in diabetic rats is significantly reduced in Roux-en-Y reconstruction after total gastrectomy [16]. Consistent with this, hyperglycemia was occasionally observed in the present case after the symptoms appeared, even though the patient was non-diabetic and had a normal preoperative blood glucose level. Therefore, it follows that reconstruction of the digestive tract increases the susceptibility to the development of hyperglycemia, which is a risk factor of disease onset.

**Table 2:** Summary of the reported cases about CTLN II developed after operation

Case	Authors (year)	Sex/age*	Operation (diagnosis)	Initial symptom	Onset**
1	Eriguchi et al. (2010) [14]	M/15	Total colectomy (severe Ulcerative colitis)	Complex partial seizure	Few days
2	Tazawa et al. (2013) [15]	F/49	Pancreaticoduodenectomy (malignant somatostatinoma)	Hepatic encephalopathy	2 years
3	Present case	M/56	Total gastorectomy (stomach cancer)	Unconsciousness	13 days

*Age when the patient underwent the operation

**Onset of the initial symptom associated with CTLN II after the operation

When several conservative treatments fail, liver transplantation, which is a practical treatment that fundamentally improves the patient’s quality of life, should be performed. Kimura et al. reported that the survival rate in CTLN2 patients treated with liver transplantation was 100%, but was 76.5% in patients who received conservative treatment [11]. Conservative treatment did not yield sufficient results in the present case, which indicated that the abdominal surgery requiring digestive tract reconstruction may exhibit resistance to conservative treatment. Liver transplantation, which is the only established radical treatment for CTLN2, will be necessary if his condition worsens; however, uncontrollable malignant tumors are a contraindication for liver transplantation. In particular, tumor recurrence or metastasis will be problematic due to his final pathological stage. Recently, a meta-analysis revealed that immunosuppressive therapies did not increase the incidence of tumor recurrence [17]. While uncontrollable malignant tumors contraindicate liver transplantation, it may be difficult to continue adjuvant chemotherapy without controlling the patient’s CTLN2 symptoms. Thus, although it is important to carefully assess the criteria and the timing, it is essential to consider treatment with liver transplantation without waiting for follow-up period of 5-year recurrence-free survival if all conservative therapies fail. Further investigation of novel conservative therapies is necessary to provide a safer, and more affordable, as an alternative to liver transplantation in the near future.

## Conclusion

Abdominal surgery requiring the reconstruction of the digestive tract alters metabolic mechanisms, such that CTLN2 symptoms may arise even in heterozygotic patients. When conservative CTLN2 treatment fails, liver transplantation, which is an established radical procedure, should be performed.

## Abbreviations

ATP: Adenosine triphosphate
CTLN II: Adult-onset type II citrullinemia
SLC25A13: Solute carrier family 25 member 13
